# Pendulous Abdomen1Read at the 37th annual meeting of the Medical Association of Central New York, held at Rochester, N. Y., October 18, 1904.

**Published:** 1905-05

**Authors:** J. P. Creveling

**Affiliations:** Auburn, N. Y.


### Pendulous Abdomen.[1]

By J. P. CREVELING, M. D., Auburn, N. Y.

THE few remarks I have to make in this paper will be confined to that flabby, sagging condition of the abdomen seen in women fairly well advanced in life, and I think in women who, in earlier years were corpulent with prominent abdomens. Observation has taught us that many individuals with large protruding abdomens, are rather spare in other parts of the body. For instance, the ankles and legs especially below the knee are small, and the upper chest about the collar bone is thin and sometimes the nates as well.

Much of such prominence is due to fat deposit, extraperitoneal, and indifferently distributed in the tissues between the skin and peritoneum. In most such cases will be seen a tendency to fatty changes in the muscle fiber, or at least such muscles become flaccid, thin and less resistant to the pressure from within. In these cases the recti muscles are usually widely separated and are thin. Pregnancy undoubtedly adds to this by subjecting the parts to continued and increasing pressure, the tension of which is largely in a lateral direction. These elements with a general visceroptosis may be sufficient to cause the deformity. The continued gravitation of the viscera I believe to be an important factor in fagging out the weakened muscles. It is not of the ordinary condition that I wish to speak today, but of the extreme, in which the abdominal wall seems to have lost all energy and the anterior abdomen hangs over the pubis like a pouch, without any apparent effort on the part of the muscles to retract and maintain it in shape.

Women with large abdominal tumors after their removal expect to return to their normal figure and genteelness, and a

---

1. Read at the 37th annual meeting of the Medical Association of Central New York, held at Rochester, N. Y., October 18, 1904.

large baggy abdomen to them is a great annoyance. It is true this condition is usually obtained, but there are times when it is not and any means calculated to remedy or improve, without too much risk, is a measure for consideration. Two cases have come under my care in which the belly wall had entirely lost tonicity and hung like a bag over the pubis. In these two cases I did an operation I have not seen advised; neither would I suggest its performance except for special reasons and when other means have failed to answer the purpose. Both these cases previous to this time had had laparatomy performed for pelvic disease. In both the general condition existed before the operation and in both the union of the incision was good. In November, 1898, a woman 58 years old entered the city hospital for the removal of an ovarian tumor. She had borne seven children, was of medium height, corpulent, the face had rather a bronzed appearance and was expressive of abdominal pain. The mammæ were large and flabby. The abdomen was large, had a boggy feel and the muscles much relaxed. She complained of a good deal of pain, the focus of which was in the right flank, but it radiated throughout the abdomen. It was made worse by walking or standing and was accompanied by a general discomfort which rendered her a semi-invalid. The bowels had been constipated and distended for years.

On examination, a cyst springing from the left ovary was discovered, not large and could not be responsible for the symptoms present. Both kidneys were in position as was the liver. A number of coils of distended intestines lay in the pouch caused by the relaxed muscles. In fact it seemed as if most of the intraperitoneal viscera were trying to find lodgement in the basin of those sagging muscles. On the following day the cavity was opened through the median line as usual, and the cyst secured, which contained only about a quart of fluid and was without special interest. The right ovary was about three times the normal size and sclerosed, while the uterus was practically normal. Most of the intestines seemed to be in the pelvis, and the stomach was found in the region of the umbilicus. The wound was closed and it united by primary union. I deluded myself in the belief that the woman would be well after a proper period of convalescence, but much to my chagrin, she appeared at my office some six months later, with an abdomen as large as before the operation, said all the old symptoms had returned and the operation had done her no good.

She had failed, however, to wear the support given her after leaving the hospital. It was then surmised that the union had been poor, the cicatrix stretched, or at best something had gone wrong in the healing process, which allowed the recti muscles to separate, the oblique to contract and thus leave the anterior abdomen without sufficient support. It was therefore determined to open the cicatrix and draw the supporting muscles forward and unite them by suture, but when the skin was cut through, the scar was found to be firm and complete, and the recti muscles could not be found even after some lateral dissection had been made. It was then decided to cut out the surperfluous belly wall, and for this purpose a semi-eliptical incision beginning just above the umbilicus and a little to the right was carried to the symphysis pubis. The maximum distance to the right of the linea alba was a little over two inches. This incision extended through the entire abdominal wall into the peritoneal cavity. A similar incision was then made on the left side, beginning and ending at the same point as that on the right. This removed something more than four inches wide of the anterior wall of the abdomen. The margins of the wound were then brought together and sutured in the usual way. Union was complete and in two weeks she was able to be out of bed. No muscle was seen during the operation, and after the wound had been closed, an incision two inches long was made, two inches to the right of the first cut, down to the peritoneum, but no muscle fiber was found. This cut, of course, was merely for inspection.

In the second case many of the general features were much the same, but the woman was younger, being but 34 years old, the mother of five children, which had been born in rapid succession. She said she had had peritonitis some three or four times, and she also had the same flabby abdomen. On examination the uterus and bladder were found prolapsed and adherent to a mass above which later proved to be the bowels. When the abdomen was opened it was discovered that the whole generative apparatus and a number of coils of intestines were stuck together in one mass, and this adhered to all the surroundings. During the disentanglement the fundus of the bladder was opened, which was sutured, and when the object of the operation had been accomplished, the uterus was suspended and the wound closed. She made a rather slow recovery. This was done in 1901 and I did not see her again until last spring, when she was referred to me for what was supposed to be post operative hernia. This proved not to be the case, but was a prolapsed condition of all the coverings of the abdomen.

The same operation as the one detailed was done, except that the incision began at the umbilicus and not above it. The result here was as satisfactory as in the case already given. In both cases, the strip of four inches was sufficient to remove all superfluous wall and restore the abdomen to normal size and shape. At present there is no manifest tendency to sagging in either patient.

22 SOUTH STREET.

